# Metabolism-related brain morphology accelerates aging and predicts neurodegenerative diseases and stroke: a UK Biobank study

**DOI:** 10.1038/s41398-023-02515-1

**Published:** 2023-06-29

**Authors:** Chenye Shen, Chaoqiang Liu, Anqi Qiu

**Affiliations:** 1 Department of Biomedical Engineering, National University of Singapore, Singapore, Singapore; 2 The N.1 Institute for Health, National University of Singapore, Singapore, Singapore; 3 NUS (Suzhou) Research Institute, National University of Singapore, Suzhou, China; 4 Institute of Data Science, National University of Singapore, Singapore, Singapore; 5 Department of Health Technology and Informatics, the Hong Kong Polytechnic University, Hung hom, Hong Kong; 6 Department of Biomedical Engineering, the Johns Hopkins University, Baltimore, MD USA

**Keywords:** Predictive markers, Physiology

## Abstract

Metabolic syndrome (MetS) is characterized by a constellation of metabolic risk factors, including obesity, hypertriglyceridemia, low high-density lipoprotein (HDL) levels, hypertension, and hyperglycemia, and is associated with stroke and neurodegenerative diseases. This study capitalized on brain structural images and clinical data from the UK Biobank and explored the associations of brain morphology with MetS and brain aging due to MetS. Cortical surface area, thickness, and subcortical volumes were assessed using FreeSurfer. Linear regression was used to examine associations of brain morphology with five MetS components and the MetS severity in a metabolic aging group (*N* = 23,676, age 62.8 ± 7.5 years). Partial least squares (PLS) were employed to predict brain age using MetS-associated brain morphology. The five MetS components and MetS severity were associated with increased cortical surface area and decreased thickness, particularly in the frontal, temporal, and sensorimotor cortex, and reduced volumes in the basal ganglia. Obesity best explained the variation of brain morphology. Moreover, participants with the most severe MetS had brain age 1-year older than those without MetS. Brain age in patients with stroke (*N* = 1042), dementia (*N* = 83), Parkinson’s (*N* = 107), and multiple sclerosis (*N* = 235) was greater than that in the metabolic aging group. The obesity-related brain morphology had the leading discriminative power. Therefore, the MetS-related brain morphological model can be used for risk assessment of stroke and neurodegenerative diseases. Our findings suggested that prioritizing adjusting obesity among the five metabolic components may be more helpful for improving brain health in aging populations.

## Introduction

Metabolic syndrome (MetS) is a constellation of conditions, including obesity, hypertriglyceridemia, low high-density lipoprotein (HDL) levels, hypertension, and hyperglycemia [1]. MetS affects 33% of the US population and increases significantly with age, reaching a prevalent rate of more than 50% in the elderly aged over 60 years [2]. Often considered a “pre-disease” state, MetS is associated with the development of cognitive impairment [3] and neurological diseases, such as stroke [4], dementia [5], and Parkinson’s disease [6] in aging populations. Given the current absence of disease-modifying treatment in the elderly [7] and the considerable burdens posed by the incidence of stroke and dementia [8, 9], there is a growing interest in understanding how to slow down or even reverse brain aging in the pre-disease stages. Consequently, determining the role of MetS in brain aging within the general population is crucial, as addressing MetS may be more beneficial for brain health. The mechanisms underlying the association between MetS and brain aging, as well as neurological diseases, remain poorly understood.

Brain morphological alterations, such as cortical thinning and volume reduction, are one of the most prominent markers of brain aging [10, 11]. Increasing evidence suggests that adults with MetS or with a diagnosis of obesity, diabetes, hypertension, high triglycerides, or low HDL have a thinner thickness in the global cortex [12, 13], frontal lobe [13–15], and central gyrus [12, 13]; and lower volumes in the whole brain [16], thalamus [12] and basal ganglia [12, 14, 17] in the healthy middle-aged and/or older populations. Nevertheless, some existing studies do not discover such associations [12–14, 18, 19]. There was also a lack of consensus on the morphological changes in the medial temporal lobe (MTL), including a hippocampal volume reduction [14, 20, 21] and MTL cortical thinning [12, 14, 21], in adults with MetS across various age and ethnic groups. Discordant results may be partly due to the use of the dichotomous diagnosis of MetS that does not capture the complexity and dimensional view of MetS and its individual components [22]. Moreover, the small sample size employed in existing studies may not well characterize the heterogeneity of brain morphology across the large age range.

While MetS is a constellation of conditions, yet there is currently a limited understanding of the relative importance of each MetS component in maintaining brain integrity. Research has suggested that multiple metabolic abnormalities often occur concurrently [1], making it difficult to disentangle their individual effects due to the complex interplay among various metabolic risk factors. In a partial least squares correlation analysis conducted on younger adults, blood pressure was found to have the least contribution to the latent variable that maximizes the covariance between cortical thickness and MetS [18]. Gaining a better understanding of this differential pattern in middle-aged to older populations is crucial, as it could inform the prioritization of preventative measures and treatment efforts against MetS-induced brain damage.

Recently, increasing attention has been paid to the estimate of brain age using MRI images via machine learning approaches [23, 24]. The gap between brain age and chronological age (brain-age gap) has been used to characterize brain aging. Patients with Alzheimer’s disease (AD) and schizophrenia exhibit a greater brain-age gap than healthy controls [25, 26]. It has been shown that the brain-age gap is correlated with a broad range of cardiovascular and metabolic risk factors [23, 24]. These findings suggested that the brain-age gap may be a good indicator of brain health. Nevertheless, few studies evaluated which metabolic component can be best used to reverse brain aging among the five metabolic components. Moreover, it is unclear whether MetS-related brain morphological abnormalities can be used to distinguish stroke and neurodegenerative diseases from metabolic brain aging.

To answer the above questions, this study leveraged the large-scale community-dwelling cohort of the UK Biobank study with broad measures, including demographics, socioeconomics, lifestyle status, MetS, and brain magnetic resonance imaging (MRI) in middle- to older-aged participants [27]. This study aimed to explore the associations of individual MetS components and the MetS overall severity with brain morphology in an aging population without major health problems (a metabolic aging group), where MetS and its components were characterized in a continuous manner and brain morphology was assessed using cortical surface area and thickness, as well as subcortical volumes. Our findings allowed the full investigation of the common and distinctive brain patterns among all the five MetS components and clarified the discrepancy in the existing findings [12–21, 28, 29]. Partial least squares (PLS) regression was employed to predict brain age based on the MetS-associated brain morphology. We expected that MetS accelerates brain aging. The more severe MetS is, the more aging the brain is. As MetS is a risk factor for neurodegenerative diseases and stroke, we expect that the PLS model can distinguish brain aging in neurodegenerative diseases and stroke from metabolic aging. This study provides direct analysis of the link among MetS, brain age, neurodegenerative diseases, and stroke.

## Methods

### Participants

**Fig. 1:**
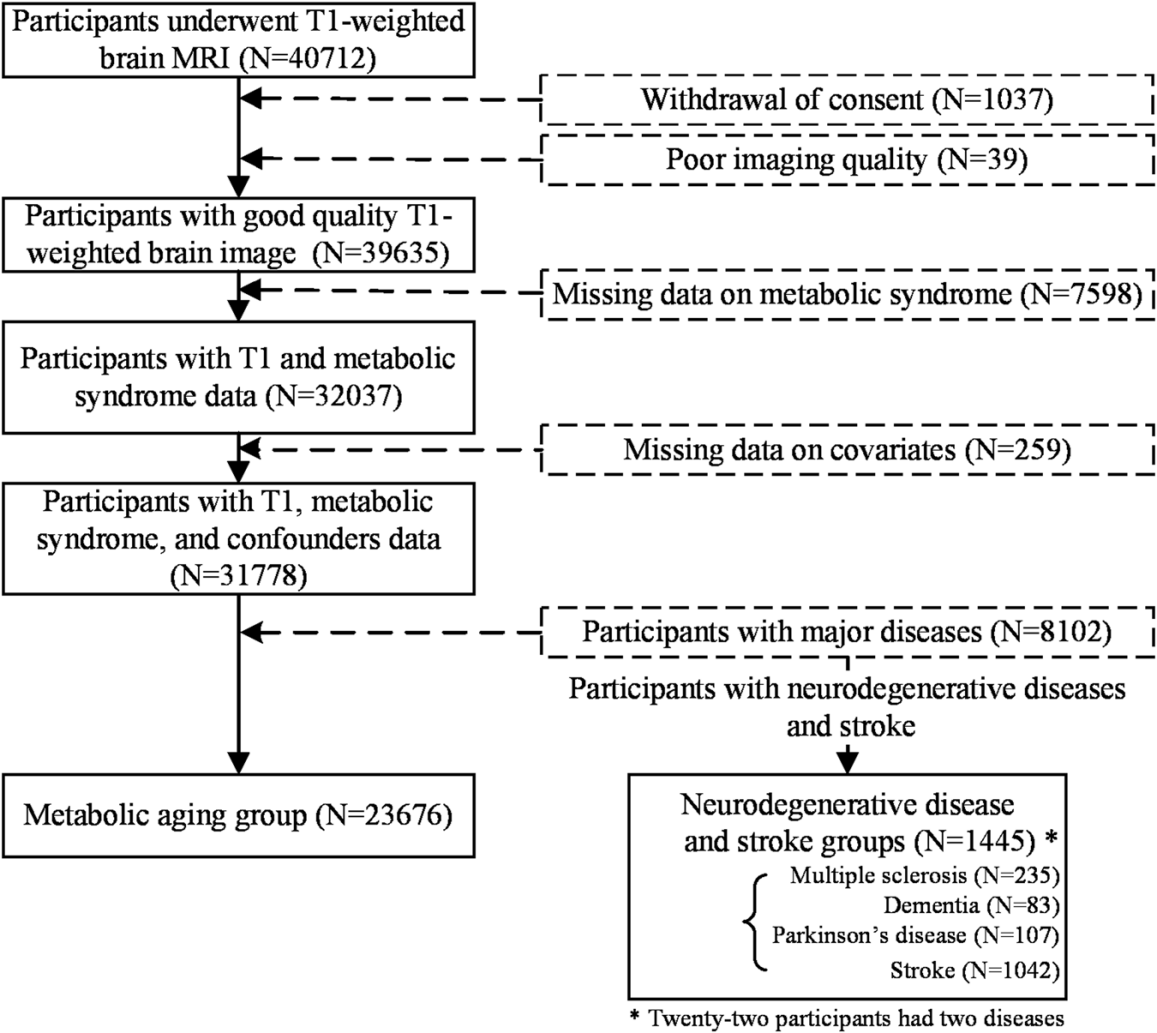
Flowchart of the participant selection for the metabolic aging group and neurodegenerative disease and stroke groups. Participants with good quality T_1_-weighted brain image, all metabolic syndrome and covariates variables were included. Of these, 23,676 with no major disease were included in the metabolic aging group, and 1445 participants with a history or current diagnosis of multiple sclerosis, dementia, Parkinson’s disease, or stroke were included in the neurodegenerative disease and stroke groups.

This study employed the sample from UK Biobank which collects biological and medical data on ~500,000 adults aged between 40 and 70 years old (https://www.ukbiobank.ac.uk). We included participants with brain MRI data (*N* = 40,712) and excluded participants because of (1) withdrawal of consent (*N* = 1037); (2) poor quality of the T_1_-weighted brain image (*N* = 39); (3) missing the data of any MetS (*N* = 7598); and (4) missing any covariate (*N* = 259), resulting 31,778 participants. This study then defined a metabolic aging group and a neurodegenerative disease group. To form a metabolic aging group, this study further excluded participants with a history or current diagnosis of cancers, cardiovascular, neurological, and psychiatric diseases (Table S1 of the Supplementary Material for details), resulting in 23,676 participants. This study formed a neurodegenerative disease and stroke group (*N* = 1445) in which participants had a history or current diagnosis of any of the following neurodegenerative diseases: multiple sclerosis (*N* = 235), dementia (*N* = 83), stroke (*N* = 1042), and Parkinson’s disease (*N* = 107). Twenty-two participants had multiple diseases. Neurodegenerative diseases and stroke were identified with reference to the algorithms developed by the UK Biobank Outcome Adjudication group [30]. Figure 1 summarizes the flowchart of the sample selection for the metabolic aging and neurodegenerative disease groups.

### Standard protocol approvals, registrations, and patient consents

Ethics approval was provided by the National Health Service, National Research Ethics Service (reference 11/NW/0382). Participants signed written informed consent and were allowed access to their health records from the UK National Health Service. This study was conducted under UK Biobank application number 57831.

### MRI acquisition and analysis

The brain MRI was acquired in three imaging centers (Cheadle, Newcastle, and Reading) using 3T Siemens Skyra scanners with a standard 32-channel RF receive head coil [31]. The T_1_-weighted image was obtained using Magnetization Prepared Rapid Acquisition Gradient-Echo (MPRAGE) with 1 × 1 × 1 mm resolution, a field-of-view of 208 × 256 × 256 mm, TI/TR/TE = 880/2000/2.01 ms, and a flip angle of 8°.

The T_1_-weighted MRI was processed through the longitudinal analysis pipeline in FreeSurfer (version 7.1.1) [32]. A post-processing quality check was conducted by one well-trained researcher based on the instruction given at https://surfer.nmr.mgh.harvard.edu/fswiki/FsTutorial/TroubleshootingData. This study extracted cortical surface area, thickness, and subcortical volumes to quantify brain morphology. The surface area and thickness were aligned voxel-wise to the FreeSurfer template. The cortical surface area and thickness were smoothed at 10 mm full width at half maximum to increase the signal-to-noise ratio and reduce the impact of misregistration.

### Metabolic syndrome (MetS)

This study included five components of MetS, including waist circumference, triglyceride, HDL, hypertension, and fasting glucose, based on the National Cholesterol Education Program Adult Treatment Panel III (NCEP‐ATP III) and International Diabetes Federation (IDF) [1]. Waist circumference was measured with a Seca 200 measuring tape after participants had removed bulky clothes. Triglyceride and HDL concentrations in the blood serum were quantified on Beckman Coulter AU5800 analyzers. Blood pressure (BP) was assessed twice using Omron 7015IT monitors, where the second reading has been suggested to be used [33]. Due to a small number of participants with fasting glucose measures, this study employed hemoglobin A1c (HbA1c) to quantify the average blood glucose (sugar) levels for the last 2 to 3 months via a Bio-Rad VARIANT II Turbo analyzer [34]. Detailed information about blood biochemistry methods and quality control procedures is available online [35].

This study also quantified the severity of metabolic syndrome (MetS severity) as the number of MetS components that fall into the NCEP‐ATP III/IDF criteria [1]: (1) elevated waist circumference (≥102 cm in males and ≥88 cm in females), (2) evaluated triglycerides (≥1.7 mmol/L or under triglyceride medication), (3) reduced HDL (<1.0 mmol/L in males and <1.3 mmol/L in females or under HDL medication), (4) elevated BP (≥130 mmHg systolic or ≥85 mmHg diastolic or under hypertension medication), (5) elevated blood glucose (HbA1c ≥42.0 mmol/mol or under diabetes treatment) [34].

### Covariates

This study selected potential covariates based on possible associations with MetS and brain morphology [36, 37]. The following covariates were included in self-reported questionnaires: demographics (age at the imaging visit, sex, ethnicity, handedness, and brain size), socioeconomic status (Townsend deprivation index, the number of years for education, and employment status), and lifestyle (smoking and alcohol consumption frequency). Ethnicity was categorized into white (97%) and non-white (3%). The brain size was estimated as brain segmentation volume via FreeSurfer. Missing data on the number of years of education were inferred from the education qualification level. This study followed the criteria defined in Davies et al. [38], that is, College or University Degree corresponds to 16 years, A levels or AS levels or equivalent is 13 years, O levels or GCSEs or equivalent, and a Certificate of Secondary Education (CSE) or equivalent corresponds to 11 years, National Vocational Qualification (NVQ) or Higher National Diploma (HND) or Higher National Certificate (HNC) or equivalent and other professional qualifications (e.g., nursing and teaching) are 10 years, Never attend school is 0 years. Employment status was classified into four groups: working, unemployed, retired, and others. Smoking status was categorized into three groups: never, former, and current. Alcohol consumption frequency was coded from never (as 0) to the most frequent (as 5). This study also included imaging sites (Cheadle, Reading, and Newcastle) as a covariate to control potential differences in brain morphology due to different scanners.

### Statistical analysis

This study compared the demographics, socioeconomics, and lifestyle characteristics in the metabolic aging group and the neurodegenerative disease and stroke group with the imaging sample. The Mann-Whitney U test and chi-squared (*χ*^2^) test were respectively used for continuous and categorical variables.

Due to the difference in the sample sizes of the metabolic aging group and the neurodegenerative disease and stroke group, this study also conducted a propensity score matching analysis using the “MatchIt” package in R (v4.2.1; R Core Team 2022) [39] to extract participants from the metabolic aging group whose demographic, socioeconomic status, and lifestyle were matched to those in the neurodegenerative groups or the stroke group. Student’s *t*-test was used to examine the difference in metabolic syndromes of the metabolic aging group with each of the neurodegenerative disease and stroke groups.

**Fig. 2:**
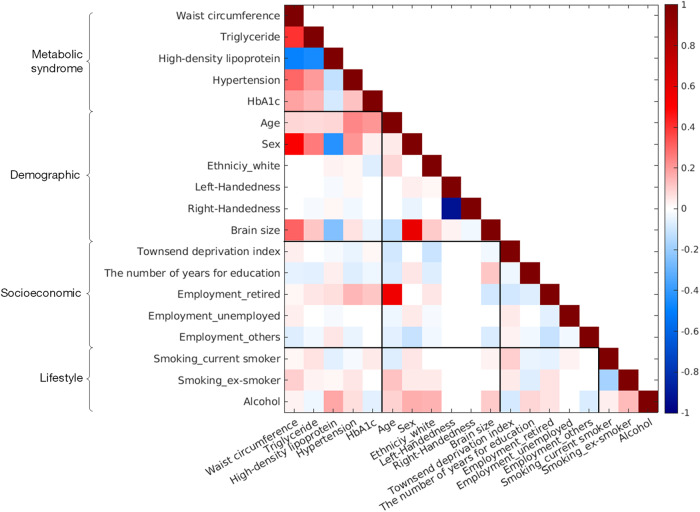
Heatmap among metabolic syndrome, demographic, socioeconomic, and lifestyle variables in the metabolic aging group. Only significant correlations are displayed (FDR-corrected *p* < 0.01). HbA1c hemoglobin A1c.

Linear regression was used to examine associations of brain morphology with individual MetS components in the metabolic aging group. Given the moderate to high correlations observed between the five MetS components (Fig. 2), we employed a two-step regression approach to evaluate the unique effect of each MetS component on brain morphology. We first regressed out the effects of the other four MetS components from the brain morphological measure (cortical surface area/cortical thickness/subcortical volumes). In the next step, the residuals from the first step served as dependent variables, and the MetS component of interest as an independent variable while controlling for the demographic, socioeconomic, and lifestyle covariates listed in Table 1.

**Table 1:** Characteristics of participants.

Characteristics	Summary		
	Imaging sample	Metabolic aging group	Neurodegenerative disease and stroke group
	(*N* = 31,778)	(*N* = 23,676)	(*N* = 1445)
**Metabolic syndrome (MetS)**			
Waist circumference (cm)	87.9 (12.5)	87.4 (12.4)^***^	90.4 (12.9)^***^
Triglyceride (mmol/L)	1.64 (0.95)	1.62 (0.94)^***^	1.76 (1.06)^***^
HDL (mmol/L)	1.48 (0.38)	1.49 (0.37)^***^	1.41 (0.36)^***^
Hypertension	61.9%	59.6%^***^	71.3%^*^
HbA1c (mmol/mol)	35.0 (5.06)	34.8 (4.95)^***^	35.6 (5.75)^***^
MetS severity	1.52 (1.19)	1.44 (1.17)^***^	1.76 (1.31)^***^
**Demographics**			
Age (years)	63.6 (7.53)	62.8 (7.45)^***^	65.5 (7.58)^***^
Sex, Male	47.5%	46.3%^**^	55.0%^***^
Ethnicity			
White	97.2%	96.9%^*^	97.6%
Non-white	2.8%	3.1%^*^	2.4%
Handedness			
Right-handedness	89.1%	89.1%	89.9%
Left-handedness	9.4%	9.5%	8.4%
Ambidexterity	1.5%	1.4%	1.7%
Brain size (cm^3^)	1193.5 (111.8)	1194.3 (112.4)	1194.8 (113.8)
**Socioeconomic**			
Townsend deprivation index	−1.91 (2.70)	−1.90 (2.71)	−1.87 (2.70)
The number of years for education (years)	13.9 (2.58)	14.0 (2.55)^***^	13.8 (2.74)^***^
Employment status			
Paid	69.3%	72.5%^***^	59%^***^
Retired	24.7%	21.5%^***^	33.2%^***^
Unemployed	1.2%	1.2%^***^	0.8%^***^
Others	4.8%	4.8%^***^	7.0%^***^
**Lifestyle**			
Smoking status			
Never smoked	61.0%	62.4%^**^	55.9%^***^
Ex-smoker	32.8%	31.5%^**^	37.0%^***^
Current smoker	6.2%	6.1%^**^	7.1%^***^
Alcohol consumption frequency	3.31 (1.39)	3.31 (1.38)	3.24 (1.47)
**Imaging site**			
Cheadle	61.5%	60.8%	65.6%^***^
Reading	13.1%	13.3%	9.9%^***^
Newcastle	25.4%	25.9%	24.5%^***^
**Neurodegenerative disease and stroke (N)**			
Multiple sclerosis			235
Dementia			83
Parkinson’s disease			107
Stroke			1042

Values are shown as mean (SD) or %. The groups are compared in two ways: first, between the imaging sample and the metabolic aging group, and second, between the metabolic aging group and the neurodegenerative disease and stroke group.

*HDL* high-density lipoprotein, *HbA1c* hemoglobin A1c.

***p < 0.001, **p < 0.01, *p < 0.05.

We also employed the same regression model to examine the associations of overall MetS risk with brain morphology, where the brain morphology measures (cortical surface area/cortical thickness/subcortical volumes) as dependent variables and the MetS severity as an independent variable while adjusting for the demographic, socioeconomic, and lifestyle covariates listed in Table 1.

These regression models were applied to each vertex on the cortex using the SurfStat toolbox (http://www.math.mcgill.ca/keith/surfstat) and subcortical volumes in MATLAB. The same sample size was 23,676 for all the statistical models. Statistical results were corrected for multiple comparisons based on random field theory (RFT) for cortical surface area and thickness and false discovery rate (FDR) for subcortical volumes at a significance level of 0.01.

We then examined which MetS component best explained brain morphology. We computed the Akaike information criterion (AIC) [40] for the above five regression models related to individual MetS components. If the deviation of the lowest AIC from others is above two, then the regression model with the lowest AIC is defined as the winning model and the corresponding MetS component is the best variable to explain brain morphology [41]. We applied this procedure at every vertex on the cortical surface and each subcortical region.

Next, we employed PLS [42] to predict the brain biological age of the metabolic aging group, where the standardized cortical and subcortical morphological measures associated with any MetS component were used as features and chronological age was the predictive variable. Ten-fold cross-validation was used to evaluate the performance of PLS. The MetS-related brain-age gap was calculated as the difference between the estimated brain age and chronological age. The chronological age was further regressed out from the brain-age gaps to adjust for age bias [43]. We employed the Kolmogorov–Smirnov (KS)-test to examine whether the brain-age gap distribution in participants with greater MetS severity is different from that in participants with less MetS severity. In other words, we tested whether greater MetS severity accelerates brain aging. FDR was used to correct statistical *p* values at a significance level of 0.05.

We then hypothesized that the MetS-related brain-age gap is larger in neurodegenerative diseases and stroke groups when compared to the metabolic aging group. The brain-age prediction model trained on the metabolic aging group was directly applied to the neurodegenerative diseases and stroke groups. Moreover, we tested whether the brain-age gap estimated based on the brain morphology associated with a specific MetS component can well distinguish a specific neurodegenerative disease or stroke from the metabolic aging group. KS-tests were used to assess the difference in the distributions of the brain-age gaps estimated by the specific-MetS-associated PLS model in the metabolic aging and specific neurodegenerative disease and stroke groups. Statistical results were corrected via FDR at a corrected *p* value of 0.05. All analyses were carried out in MATLAB R2017b (The MathWorks, Inc., USA).

## Results

### Demographics

Table 1 lists the demographic, socioeconomic, and lifestyle characteristics in the whole imaging sample, the metabolic aging, and neurodegenerative disease groups. The metabolic measures were acquired in the first visit to the UK Biobank study, while brain images used in this study were acquired 8.8 ± 1.7 years later. Compared to the whole imaging sample, the metabolic aging group was slightly younger, metabolically healthier, more educated (all *p* < 0.001), and more likely to be female (*p* = 0.004), non-white (*p* = 0.04), employed (*p* < 0.001), and non-smokers (*p* = 0.013). Participants in the neurodegenerative disease and stroke group were older, more metabolically unhealthy, less educated, and more likely to be male, retired, and smokers either in the past or present (all *p* < 0.001, except for hypertension status, *p* = 0.03) when compared to the metabolic aging group. The proportion of participants in the three imaging sites was different between the metabolic aging group and neurodegenerative disease and stroke groups (*p* < 0.001).

When matching the demographic, socioeconomic, lifestyle variables, and the sample size, participants in the neurodegenerative disease groups (dementia, Parkinson’s disease, multiple sclerosis) and the stroke group exhibited significantly higher metabolic severity (*p* < 0.05) (Table S3–S6 in the Supplementary Material).

### Associations of metabolic syndromes with brain morphology

**Fig. 3:**
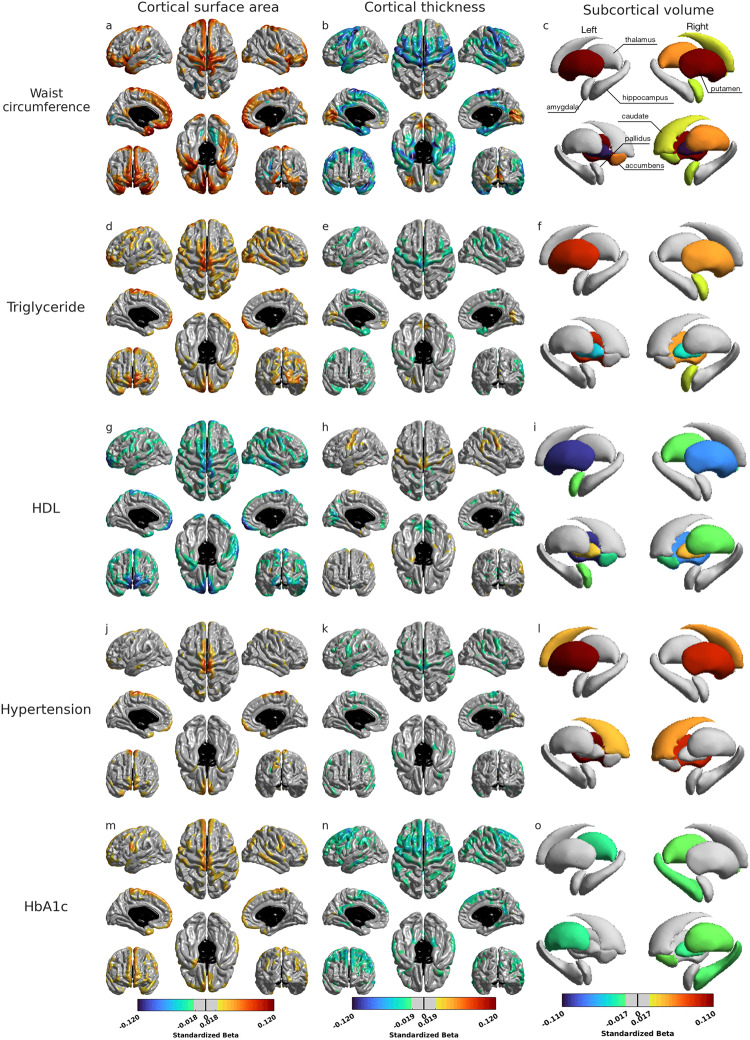
Statistical maps for the associations of metabolic syndromes with cortical surface area (left column), cortical thickness (middle column), and subcortical volumes (right column) in the metabolic aging group. Rows from top to bottom display the association with waist circumference (**a**–**c**), triglyceride (**d**–**f**), high-density lipoprotein (HDL) (**g**–**i**), hypertension (**j**–**l**), and hemoglobin A1c (HbA1c) (**m**–**o**), respectively. The results were adjusted for age at the MRI visit, sex, Townsend deprivation index, ethnicity, age completed full-time education, smoking status, alcohol consumption frequency, employment status, brain size, and imaging sites. Significant brain regions are displayed at the corrected *p* value <0.01 for cortical morphology. L left, R right, Acc accumbens, Amyg amygdala, Caud caudate, Hipp hippocampus, Pall pallidum, Put putamen, Thal thalamus. **p < 0.001, *p < 0.01.

Figure 3 shows the significant associations of each MetS with cortical surface area, thickness, and subcortical volumes. For all five MetS components, a consistent pattern emerged in the central and superior frontal gyri, whereby worse MetS status was associated with increased cortical surface area and decreased thickness. Nevertheless, there were distinct associations of brain morphology with individual MetS components.

Higher waist circumference was additionally associated with larger cortical surface area and thinner cortical thickness in the lateral frontal lobe, supramarginal gyrus, lateral temporal lobe, posterior cingulate cortex, and insula. The thickness in the medial temporal lobe also decreased with increasing waist circumference (Fig. 3b). Moreover, greater waist circumference was associated with the larger bilateral putamen, right caudate, and thalamus, as well as right amygdala (Fig. 3c).

The triglyceride and HDL had similar effects on brain morphology. Greater triglyceride or lower HDL was associated with increased cortical surface area and decreased cortical thickness in the bilateral central gyrus, supramarginal gyrus, and middle temporal gyrus (Fig. 3d, e, g, h). The putamen and pallidus showed significant associations (Fig. 3f, i). However, only the bilateral nucleus accumbens and the right thalamus were associated with HDL but not with triglyceride.

Hypertension had the least effects on brain morphology compared to the other four metabolic syndromes. Beyond the common pattern, greater hypertension was related to reduced thickness in the parahippocampal gyrus, posterior cingulate cortex, and insula, as well as to an increased volume in the bilateral caudate and putamen (Fig. 3k, l).

The effect of HbA1c was much stronger than that of hypertension and dyslipidemia, but weaker than that of waist circumference. Higher HbA1c was significantly associated with larger cortical surface area and thinner cortical thickness in the lateral orbitofrontal cortex, frontal gyrus, motor region, lateral temporal lobe, and superior and inferior parietal cortex. The association between higher HbA1c and thinner cortex spread to the posterior cingulate cortex, medial temporal lobe, and precuneus (Fig. 3m, n). Our findings also showed HbA1c-associated thalamus, pallidus, and hippocampus volume reductions (Fig. 3o).

### Associations of the MetS severity with brain morphology

**Fig. 4:**
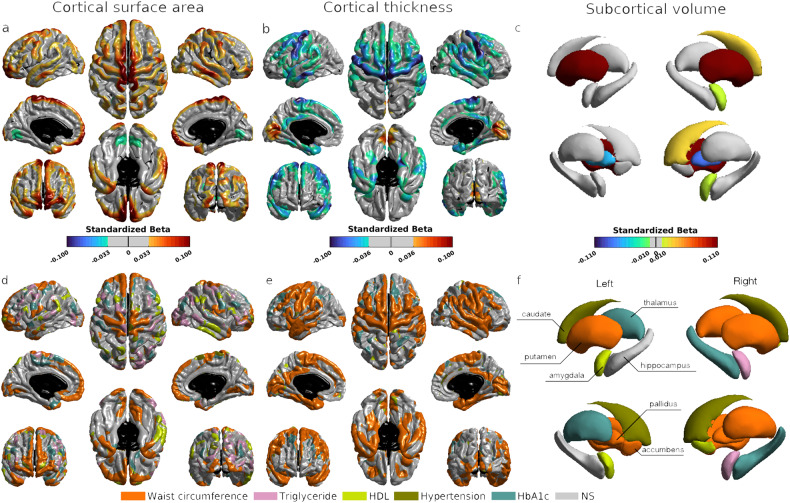
Summary of associations between the metabolic syndrome and brain morphology. Panels (**a**)–(**c**) illustrate the statistical maps for the associations between the metabolic syndrome severity (MetS severity) and cortical surface area, cortical thickness and subcortical volumes, respectively. Panels (**d**)–(**f**) shows the metabolic syndrome component that most contributed to cortical surface area, cortical thickness and subcortical volumes, respectively. Significant brain regions are displayed at the corrected *p* value <0.01 for cortical morphology. HDL high-density lipoprotein, HbA1c hemoglobin A1c, L left, R right. Acc accumbens, Amyg amygdala, Caud caudate, Hipp hippocampus, Pall pallidus, Put putamen, Thal thalamus. **corrected *p* < 0.001, *corrected *p* < 0.01.

The statistical maps between the MetS severity and cortical morphology (Fig. 4a, b) were similar to the consistent pattern observed in individual MetS components (Fig. 3). That is, greater MetS severity was associated with increased cortical surface area and decreased thickness in the central and superior frontal gyri. Moreover, elevated MetS severity was related to cortical surface area expansion and thickness reduction in the lateral temporal lobe, posterior cingulate cortex, insula, and medial temporal lobe. Furthermore, increased severity of MetS was associated with an increased volume in the right amygdala and caudate, and bilateral putamen, as well as with a reduced volume in the pallidus, where the strongest effects were in the bilateral putamen and pallidus (Fig. 4c).

### The most contributed MetS component to brain morphology

All the above statistical analyses were run on the same sample of 23,676. This study employed AIC to examine which MetS component most contributed to brain morphology. Figure 4d–f illustrates the representative MetS that most contributed to the cortical surface area, thickness, and subcortical volumes. Waist circumference best explained the considerable variance in most of the MetS-associated cortical regions, thalamus, putamen, and pallidus. HbA1c explained the frontal and supramarginal thickness and right hippocampus volume. Dyslipidemia played a role in explaining the surface area in the frontal, parietal, and lateral medial cortex and the amygdala volume. HDL was most associated with the temporal cortical surface area, left amygdala, and accumbens volumes. Among the five metabolic symptoms, hypertension had the least influence on brain morphology, except on the caudate volume.

### Metabolic syndrome accelerates brain aging

**Fig. 5:**
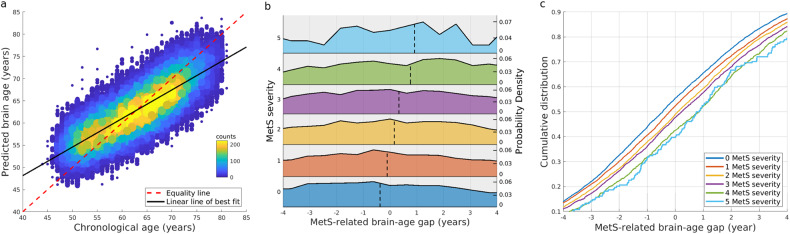
Brain age prediction. Panel (**a**) illustrates the scatterplot of the chronological age and the predicted brain age. Each dot represents one participant. The color of the dots represents the number of participants in that location. Panel (**b**) shows the brain-age gap distribution in terms of the MetS severity. The dashed line indicates the mean of each distribution. Panel (**c**) shows the cumulative distribution of the MetS-related brain-age gap at each MetS severity level.

When combining all brain morphological features associated with any one of the five MetS components, MetS-related brain morphology accurately predicted chronological age in the metabolic aging group (correlation *r* = 0.80; Fig. 5a). A root-mean-square error was 4.45 years and a mean absolute error was 3.56 years.

Figure 5b, c respectively illustrate the probability and cumulative distributions of the brain-age gaps at each MetS severity level. The mean brain-age gaps were −0.37 ± 3.45, −0.11 ± 3.57, 0.16 ± 3.59, 0.33 ± 3.62, 0.76 ± 3.57, 0.91 ± 3.64 years as increasing the MetS severity from 0 to 5, respectively. KS-tests showed significantly increasing brain-age gaps from no MetS to the MetS level of 1 (KS-test = 0.039, FDR-corrected *p* < 0.001), between the MetS levels of 1 and 2 (KS-test = 0.038, FDR-corrected *p* < 0.001), and between the MetS levels of 3 and 4 (KS-test = 0.062, FDR-corrected *p* = 0.005). But, there was no statistically significant difference in the distribution of the brain-age gap between the MetS severity levels of 2 and 3 (KS-test = 0.027, FDR-corrected *p* = 0.11) and between the MetS severity levels of 4 and 5 (KS-test = 0.048, FDR-corrected *p* = 0.82). These results suggested the acceleration of brain aging due to the elevated MetS severity.

### MetS-related brain-age gap predicts neurodegenerative diseases and stroke

The PLS model that was trained based on all MetS-associated brain morphology in the metabolic aging group estimated greater brain-age gaps for participants with dementia (2.44 ± 5.30 years, KS-test = 0.23, FDR-corrected *p* < 0.001), multiple sclerosis (1.94 ± 5.09 years, KS-test = 0.21, FDR-corrected *p* < 0.001), Parkinson’s disease (1.28 ± 4.19 years, KS-test = 0.17, FDR-corrected *p* = 0.006), and stroke (0.47 ± 4.00, KS-test = 0.07, FDR-corrected *p* < 0.001), compared with the metabolic aging group. These findings suggested that the MetS-related brain-age gap can be a good indicator of neurodegenerative diseases and stroke.

**Fig. 6:**
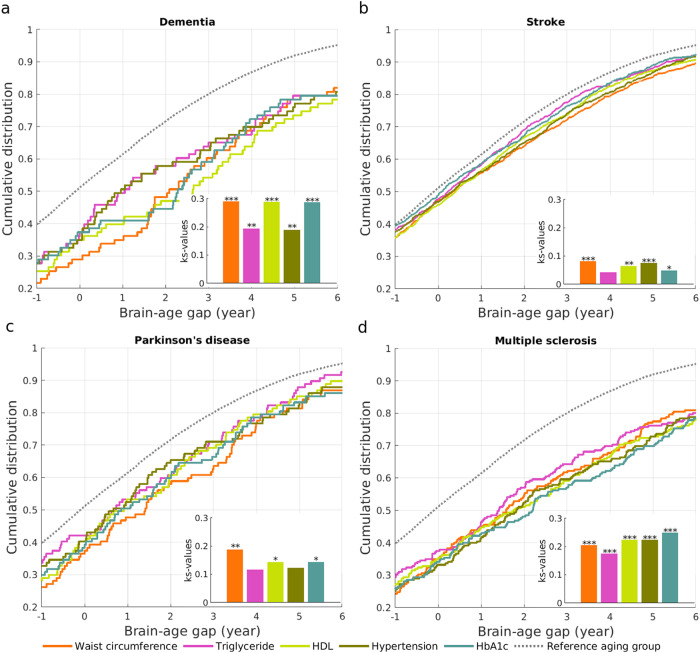
Cumulative distributions of brain-age gaps in the neurodegenerative disease groups based on the brain morphology associated with each metabolic syndrome (MetS). Panels (**a**)–(**d**) show the cumulative distributions of brain-age gaps in the dementa, stroke, Parkinson’s disease, and multiple sclerosis group, respectively. The brain-age gaps were predicted using all brain morphology that were associated with any one of the five MetS components. The dashed line represents the brain-age gaps in the aging group based on the brain morphology associated with overall MetS. Kolmogorov–Smirnov test was used to verify whether there was a significant difference in the cumulative distributions between the brain-age gap in each neurodegenerative disease group and the aging group. Abbreviations: HDL, high-density lipoprotein; HbA1c, hemoglobin A1c. ***corrected *p* < 0.001, **corrected *p* < 0.01, *corrected *p* < 0.05.

When the PLS regression only employed the brain morphology identified by individual MetS, the brain-age gap for dementia was much larger than that in the metabolic aging group (see Fig. 6a, all FDR-corrected *p* < 0.01). The largest difference in the brain-age gap occurred when using the PLS regression learned from the brain morphology associated with weight circumference (KS-test = 0.29, FDR-corrected *p* < 0.001), HDL (KS-test = 0.29, FDR-corrected *p* < 0.001), and HbA1c (KS-test = 0.29, FDR-corrected *p* < 0.001). Similarly, waist circumference and hypertension-related PLS regressions showed the largest deviation of the brain-age gap in the stroke group from the metabolic aging group (see Fig. 6b), and waist circumference-related brain-age gaps best distinguish the Parkinson’s disease group (see Fig. 6c). The PLS regressions of all MetS components showed the largest deviation of the brain-age gap in the multiple sclerosis group from the metabolic aging group (see Fig. 6e). The detailed KS-test values and corresponding *p* values are reported in Table S2 of the Supplementary Material.

## Discussion

This study capitalized on the brain structural images and clinical data from the UK Biobank and explored the associations of the five metabolic components with the brain morphology, such as cortical surface area, thickness, and subcortical volumes. Our findings suggested that widespread cortical morphology, particularly in the frontal, temporal, sensorimotor cortex, and basal ganglia were commonly associated with the five metabolic components. The elevated MetS severity accelerated brain aging. Moreover, our findings demonstrated that the MetS-related brain-age gap can well distinguish stroke and neurodegenerative diseases from aging but it does not specify the type of these diseases. Therefore, our findings to some degree supported that the MetS-related brain morphological model can be used as a risk assessment for stroke and neurodegenerative diseases.

This study conducted a comprehensive analysis of the five metabolic components and brain morphological measures based on a large sample size of the aging population without major illness. Our findings highlighted the cortical surface area and thickness of the frontal lobe, sensorimotor region, and temporal lobe, as well as basal ganglia volumes commonly in association with all five metabolic symptoms. These findings were largely consistent with existing findings related to the MetS dichotomous diagnosis [13, 14]. Individuals with greater MetS severity experienced faster brain aging. Those at the greatest severity of MetS had an average 1-year larger brain-age gap than those without risk. According to the NCEP‐ATP III/IDF definition, only those who met three or more criteria are considered to have MetS [1], while our results showed that attention should be drawn even when one or two metabolic problems were present. In particular, obesity assessed by waist circumference showed the strongest association with the widespread brain morphology among the five metabolic components even after controlling for the whole brain volume. A recent study has shown that older brain age associated with obesity and poor metabolic components can be reversed following bariatric surgery-induced weight loss. The overall effect seemed to be driven by a global change across all brain regions and not from a specific region [44]. Hence, our findings provided further evidence that prioritizing adjusting obesity among the five metabolic components may be more helpful for improving brain health in aging populations.

We discovered significant correlations between the MetS severity and the volumes of the right caudate and amygdala, in line with previous large-scale studies that showed increased volumes of these structures in association with obesity [28, 45–47]. Furthermore, the distinct patterns of the brain morphological associations with the five individual MetS components mainly occur in the subcortical and cortical basal regions, particularly the basal ganglia, amygdala, and orbitofrontal cortex. These structures have been implicated in food-related reward circuits [48, 49]. Excessive stimulation of these circuits has been proposed to contribute to overeating and is associated with obesity and the other MetS components [14, 50]. It is unclear whether these circuits play a crucial role in differentiating the associations of individual MetS components with brain morphology. However, it is possible that the increase in the amygdala and caudate volumes may compensate for the cortical atrophy of these reward circuits.

Our results also suggest a lateralization effect of the caudate and amygdala with the MetS severity, which persisted even after adjusting for handedness. However, the evidence for lateralization in the food appetite network is inconclusive, with some studies indicating a left preference [51, 52] and others pointing to a right tendency [53, 54]. Given only two subcortical regions exhibited a significant lateralization effect related to the MetS severity in our study, we advise against drawing strong conclusions regarding the lateralization effect in the context of MetS.

Additionally, we identified two physiologically adjacent regions, the putamen and pallidum, that displayed opposite relationships with the MetS severity. A previous UK Biobank study also found contrasting associations between the putamen and pallidum volumes with obesity [47]. However, the biological significance of these opposite relationships remains unclear.

Our study suggested that the MetS-associated brain morphological features can be considered as an indicator of brain aging. That is, individuals with the most MetS severity had a greater brain-age gap than those without MetS. The PLS model, trained based on the MetS-associated brain features in the relatively aging population, can be directly applied to stroke and neurodegenerative diseases, such as dementia, Parkinson’s disease, and multiple sclerosis, in the UK Biobank study. The PLS model can identify patients with these neurodegenerative diseases and stroke with a greater brain-age gap than that in the metabolic aging group. These findings can also be supported by the thinning in the medial temporal lobe, including the entorhinal cortex and parahippocampal cortex, temporal pole, and posterior cingulate cortex, associated with waist circumference and the severity of MetS and the hippocampal volume reduction associated with HbA1c and the severity of MetS. Previous histological and imaging studies have shown that the volume reduction in the entorhinal cortex, parahippocampal gyrus, and hippocampus is pathologically associated with early AD [55, 56]. Indeed, the brain features related to waist circumference, HbA1c, and the severity of MetS can well distinguish AD from the aging group. Hence, our findings provided neural support that obesity, diabetes, and MetS were associated with an increased risk of AD [5, 57, 58]. Similarly, our findings suggested that waist circumference most contributed to a volumetric reduction in the basal ganglia, a hallmark of Parkinson’s disease [59]. The brain morphology related to waist circumference well distinguished Parkinson’s disease from aging. Likewise, the brain morphology associated with individual five MetS components predicted greater brain-age gaps in stroke and multiple sclerosis than aging. By comparisons, several studies have measured the brain age of patients with brain disorders and found that the brain-age gap in schizophrenia was on average 3 years larger, that in mild cognitive impairment and AD was on average 6 and 10 years larger, respectively [25, 26]. Notably, individuals with neurodegenerative diseases or stroke also experienced a higher metabolism than healthy individuals. Hence, the MetS-associated brain morphology is sensitive to detecting stroke and neurodegenerative diseases but not specific to any type of disease.

Several limitations are worth noticing. First, this study was cross-sectional. The longitudinal analysis would be crucial to understanding the trajectory of the MetS influence on brain morphology in aging. Second, the UK Biobank imaging sample lives in less deprived areas and is healthier than the wider UK population [60–62], which may limit generalizability. Last but not least, this study limited the analysis to brain morphology. The UK Biobank study also provides other brain MRI modalities, including functional MRI and diffusion MRI [27], which need further investigation of MetS effects on brain functional and structural organization.

In conclusion, the five key metabolic syndromes significantly affected widespread brain morphology and elevated brain aging in the aging population. The MetS-related morphology well predicted elevated brain aging in stroke and neurodegenerative diseases, suggesting its role in estimating the risk of individuals. Our study suggested that prevention and timely treatment of metabolic syndromes, especially abdominal obesity, is needed for improving brain health.

## Supplementary information

Supplementary material

## Data Availability

All bona fide researchers can apply to access the UK Biobank research resource to conduct health-related research that is in the public interest.

## Code availability

The code is available upon request from the authors.
