# Supplementary material for: Metabolism-related brain morphology accelerates aging and predicts neurodegenerative diseases and stroke: a UK Biobank study
Source: Transl Psychiatry. 2023 Jun 29;13:233. doi: 10.1038/s41398-023-02515-1 (PMC10310693; doi:10.1038/s41398-023-02515-1)
Supplement: Supplementary file 1 — Supplementary material [file 41398_2023_2515_MOESM1_ESM.docx]

**Supplementary Materials**

**Table S1.** Exclusion criteria for the metabolic aging group.

| **Source** | **Conditions** | **Code** |
| --- | --- | --- |
| **Self-reported (UK Biobank Field ID 20002, 134)** | Dementia/alzheimers/cognitive impairment | 1263 |
|  | Parkinson’s disease | 1262 |
|  | Chronic degenerative neurological problem | 1258 |
|  | Acute infective polyneuritis/guillain-barre syndrome | 1256 |
|  | Multiple Sclerosis | 1261 |
|  | Other demyelinating disease | 1397 |
|  | Stroke or ischemic stroke | 1081 |
|  | Brain hemorrhage | 1491 |
|  | Brain/intracranial abscess | 1245 |
|  | Cerebral aneurysm | 1425 |
|  | Cerebral palsy | 1433 |
|  | Encephalitis | 1246 |
|  | Epilepsy | 1264 |
|  | Head injury | 1266 |
|  | Infections of the nervous system | 1244 |
|  | Ischemic stroke | 1583 |
|  | Meningioma (benign) | 1659 |
|  | Meningitis | 1247 |
|  | Motor Neuron Disease | 1259 |
|  | Neurological injury/trauma | 1240 |
|  | Spina bifida | 1524 |
|  | Subdural hematoma | 1083 |
|  | Subarachnoid hemorrhage | 1086 |
|  | Transient ischemic attack | 1082 |
|  | Any cancer |  |
| **Health record (UK Biobank Field ID 41271, 41270, 40001, 40002, 40009)** | Sporadic Creutzfeldt-Jakob disease | ICD-10: A81.0 |
|  | Inflammatory diseases of the central nervous system | ICD-9: 320-327 ICD-10: G00-G09 |
|  | Systemic atrophies primarily affecting the central nervous system | ICD-10: G10-G14 |
|  | Extrapyramidal and movement disorders | ICD-10: G20-G26 |
|  | Other degenerative diseases of the nervous system | ICD-10: G30-G32 |
|  | Demyelinating diseases of the central nervous system | ICD-10: G35-G37 |
|  | Multi-system degeneration of the autonomic nervous system | ICD-10: G90.3 |
|  | Hereditary and degenerative diseases of the central nervous system | ICD-9: 330-337 |
|  | Organic psychotic conditions | ICD-9: 290-294 |
|  | Ischemic heart diseases | ICD-9: 410-414  ICD-10: I20-I25 |
|  | Cerebrovascular diseases | ICD-9: 430-438 ICD-10: I60-I69 |
|  | Any cancer |  |

**Table S2.** Kolmogorov-Smirnov (KS) test statistics and their corresponding p-values. The KS tests were performed on the cumulative distributions of brain-age gaps in the neurodegenerative disease groups based on the brain morphology associated with each metabolic syndrome (MetS) and in the metabolic aging group.

|  | **Waist circumference** | | **Triglyceride** | | **HDL** | | **Hypertension** | | **HbA1c** | |
| --- | --- | --- | --- | --- | --- | --- | --- | --- | --- | --- |
|  | KS-test | p-value | KS-test | p-value | KS-value | p-value | KS-value | p-value | KS-value | p-value |
| **Dementia** | 0.29 | **< .001** | 0.19 | **.008** | 0.29 | **< .001** | 0.19 | **.009** | 0.29 | **< .001** |
| **Stroke** | 0.08 | **< .001** | 0.04 | .081 | 0.06 | **.001** | 0.07 | **< .001** | 0.05 | **.030** |
| **Parkinson’s disease** | 0.19 | **.002** | 0.12 | .127 | 0.14 | **.036** | 0.12 | .097 | 0.14 | **.036** |
| **Motor neuron disease** | 0.25 | .062 | 0.24 | .084 | 0.28 | **.035** | 0.29 | **.020** | 0.26 | .050 |
| **Multiple sclerosis** | 0.20 | **< .001** | 0.18 | **< .001** | 0.22 | **< .001** | 0.22 | **< .001** | 0.25 | **< .001** |
| **Huntington’s disease** | 0.16 | .928 | 0.24 | .594 | 0.29 | .354 | 0.19 | .841 | 0.24 | .594 |

**Table S3.** Characteristics of the dementia and metabolic aging groups. The two groups are matched in terms of demographic, socioeconomic, lifestyle variables, and the sample size.

| **Characteristics** | **Summary** | | ***P* value** |
| --- | --- | --- | --- |
|  | **Matched metabolic aging group**  **(N=83)** | **Dementia group**  **(N=83)** |  |
| **Metabolic syndrome (MetS)** | | | |
| Waist circumference (cm) | 86.9 (11.4) | 91.2 (12.5) | .04* |
| Triglyceride (mmol/L) | 1.57 (0.81) | 1.99 (1.23) | .02* |
| HDL (mmol/L) | 1.46 (0.32) | 1.38 (0.38) | .24 |
| Hypertension | 62.7% | 70.1% | .36 |
| HbA1c (mmol/mol) | 35.0 (5.32) | 36.0 (5.66) | .28 |
| MetS severity | 1.45 (1.23) | 1.99 (1.17) | .01* |
| **Demographics** | | | |
| Age (years) | 68.0 (6.64) | 67.8 (6.70) | .89 |
| Sex, Male | 59.7% | 61.2% | .86 |
| Ethnicity | | | |
| White | 98.8% | 98.8% | .99 |
| Non-white | 1.2% | 1.2% |  |
| Handedness |  |  |  |
| Right-handedness | 89.1% | 91.6% |  |
| Left-handedness | 9.5% | 8.4% | .58 |
| Ambidexterity | 1.4% | 0.0% |  |
| **Socioeconomic** | | | |
| Townsend deprivation index | -2.02 (2.38) | -1.94 (2.87) | .86 |
| The number of years for education (years) | 13.7 (2.55) | 13.6 (2.56) | .79 |
| Employment status | | | |
| Paid | 50.6% | 50.6% | .99 |
| Retired | 45.8% | 45.8% |  |
| Unemployed | 0.0% | 0.0% |  |
| Others | 3.6% | 3.6% |  |
| **Lifestyle** | | | |
| Smoking status | | | |
| Never smoked | 55.4% | 53.0% | .95 |
| Ex-smoker | 38.6% | 41.0% |  |
| Current smoker | 6.0% | 6.0% |  |
| Alcohol consumption frequency | 3.61 (1.30) | 3.33 (1.58) | .26 |

Abbreviations: HDL, high density lipoprotein; HbA1c, hemoglobin A1c.

Values are shown as mean (SD) or %. *P* values are fdr corrected.

*** p < .001, ** p < .01, * p < .05.

**Table S4.** Characteristics of the Parkinson’s disease and metabolic aging groups. The two groups are matched in terms of demographic, socioeconomic, lifestyle variables, and the sample size.

| **Characteristics** | **Summary** | | ***P* value** |
| --- | --- | --- | --- |
|  | **Matched metabolic aging group**  **(N=107)** | **Parkinson’s disease group**  **(N=107)** |  |
| **Metabolic syndrome (MetS)** | | | |
| Waist circumference (cm) | 86.7 (10.5) | 90.2 (12.0) | .04* |
| Triglyceride (mmol/L) | 1.59 (0.92) | 1.67 (0.86) | .53 |
| HDL (mmol/L) | 1.53 (0.34) | 1.45 (0.35) | .15 |
| Hypertension | 59.0% | 73.5% | .05 |
| HbA1c (mmol/mol) | 35.0 (6.10) | 35.1 (6.53) | .85 |
| MetS severity | 1.22 (1.05) | 1.69 (1.18) | <.01** |
| **Demographics** | | | |
| Age (years) | 68.9 (7.50) | 68.7 (7.49) | .86 |
| Sex, Male | 59.0% | 60.2% | .88 |
| Ethnicity | | | |
| White | 100.0% | 99.1% | .32 |
| Non-white | 0.0% | 0.8% |  |
| Handedness |  |  |  |
| Right-handedness | 88.8% | 86.9% |  |
| Left-handedness | 9.3% | 8.4% | .51 |
| Ambidexterity | 1.9% | 4.7% |  |
| **Socioeconomic** | | | |
| Townsend deprivation index | -1.72 (2.71) | -2.00 (2.37) | .21 |
| The number of years for education (years) | 13.4 (2.66) | 13.6 (2.87) | .82 |
| Employment status | | | |
| Paid | 45.8% | 44.9% | .81 |
| Retired | 50.5% | 49.5% |  |
| Unemployed | 0.0% | 0.0% |  |
| Others | 3.7% | 5.6% |  |
| **Lifestyle** | | | |
| Smoking status | | | |
| Never smoked | 62.6% | 62.6% | .94 |
| Ex-smoker | 34.6% | 33.7% |  |
| Current smoker | 2.8% | 3.7% |  |
| Alcohol consumption frequency | 3.71 (1.25) | 3.42 (1.42) | .17 |

Abbreviations: HDL, high density lipoprotein; HbA1c, hemoglobin A1c.

Values are shown as mean (SD) or %. *P* values are fdr corrected.

*** p < .001, ** p < .01, * p < .05.

**Table S5**. Characteristics of the multiple sclerosis and metabolic aging groups. The two groups are matched in terms of demographic, socioeconomic, lifestyle variables, and the sample size.

| **Characteristics** | **Summary** | | ***P* value** |
| --- | --- | --- | --- |
|  | **Matched metabolic aging group**  **(N=235)** | **Multiple sclerosis group**  **(N=235)** |  |
| **Metabolic syndrome (MetS)** | | | |
| Waist circumference (cm) | 85.9 (12.6) | 86.1 (13.4) | .86 |
| Triglyceride (mmol/L) | 1.52 (0.84) | 1.67 (1.13) | .13 |
| HDL (mmol/L) | 1.53 (0.39) | 1.44 (0.35) | .02* |
| Hypertension | 53.7% | 61.7% | .12 |
| HbA1c (mmol/mol) | 34.7 (5.17) | 34.6 (4.26) | .87 |
| MetS severity | 1.29 (1.14) | 1.55 (1.21) | .03* |
| **Demographics** | | | |
| Age (years) | 61.3 (7.73) | 61.5 (7.61) | .80 |
| Sex, Male | 39.4% | 38.3% | .83 |
| Ethnicity | | | |
| White | 94.9% | 97.9% | .10 |
| Non-white | 5.1% | 2.1% |  |
| Handedness |  |  |  |
| Right-handedness | 89.0% | 89.8% |  |
| Left-handedness | 9.6% | 9.4% | .89 |
| Ambidexterity | 1.4% | 0.8% |  |
| **Socioeconomic** | | | |
| Townsend deprivation index | -1.92 (2.47) | -2.03 (2.37) | .35 |
| The number of years for education (years) | 14.2 (2.55) | 14.1 (2.50) | .82 |
| Employment status | | | |
| Paid | 70.6% | 70.6% | .87 |
| Retired | 20.8% | 20.4% |  |
| Unemployed | 2.6% | 1.7% |  |
| Others | 6.0% | 7.3% |  |
| **Lifestyle** | | | |
| Smoking status | | | |
| Never smoked | 63.0% | 63.0% | .99 |
| Ex-smoker | 29.4% | 29.4% |  |
| Current smoker | 7.6% | 7.6% |  |
| Alcohol consumption frequency | 3.28 (1.41) | 3.01 (1.56) | .08 |

Abbreviations: HDL, high density lipoprotein; HbA1c, hemoglobin A1c.

Values are shown as mean (SD) or %. *P* values are fdr corrected.

*** p < .001, ** p < .01, * p < .05.

**Table S6.** Characteristics of the stroke and metabolic aging groups. The two groups are matched in terms of demographic, socioeconomic, lifestyle variables, and the sample size.

| **Characteristics** | **Summary** | | ***P* value** |
| --- | --- | --- | --- |
|  | **Matched metabolic aging group**  **(N=1042)** | **Stroke group**  **(N=1042)** |  |
| **Metabolic syndrome (MetS)** | | | |
| Waist circumference (cm) | 89.3 (11.9) | 91.2 (12.8) | <.01** |
| Triglyceride (mmol/L) | 1.64 (0.93) | 1.76 (1.03) | .02* |
| HDL (mmol/L) | 1.48 (0.38) | 1.40 (0.36) | <.001*** |
| Hypertension | 65.5% | 74.5% | <.001*** |
| HbA1c (mmol/mol) | 35.2 (4.73) | 35.8 (6.04) | .03* |
| MetS severity | 1.55 (1.16) | 1.94 (1.20) | <.001*** |
| **Demographics** | | | |
| Age (years) | 66.3 (7.50) | 66.2 (7.49) | .79 |
| Sex, Male | 57.1% | 58.2% | .65 |
| Ethnicity | | | |
| White | 97.9% | 97.4% | .51 |
| Non-white | 2.1% | 2.6% |  |
| Handedness |  |  |  |
| Right-handedness | 89.1% | 91.8% |  |
| Left-handedness | 9.5% | 6.9% | .09 |
| Ambidexterity | 1.4% | 1.3% |  |
| **Socioeconomic** | | | |
| Townsend deprivation index | -1.89 (2.73) | -1.90 (2.71) | .32 |
| The number of years for education (years) | 13.7 (2.62) | 13.7 (2.69) | .79 |
| Employment status | | | |
| Paid | 58.5% | 58.4% | .63 |
| Retired | 34.6% | 34.6% |  |
| Unemployed | 0.9% | 0.5% |  |
| Others | 6.0% | 6.5% |  |
| **Lifestyle** | | | |
| Smoking status | | | |
| Never smoked | 54.5% | 55.0% | .40 |
| Ex-smoker | 39.0% | 38.5% |  |
| Current smoker | 6.5% | 6.5% |  |
| Alcohol consumption frequency | 3.34 (1.41) | 3.26 (1.43) | .25 |

Abbreviations: HDL, high density lipoprotein; HbA1c, hemoglobin A1c.

Values are shown as mean (SD) or %. *P* values are fdr corrected.

*** p < .001, ** p < .01, * p < .05.
